# Patient Organizations’ Digital Responses to the COVID-19 Pandemic: Scoping Review

**DOI:** 10.2196/58566

**Published:** 2024-12-20

**Authors:** Simon Wallraf, Marie-Luise Dierks, Cosima John, Jonas Lander

**Affiliations:** 1 Institute for Epidemiology, Social Medicine and Health Systems Research Hannover Medical School Hanover Germany

**Keywords:** patient organizations, COVID-19, digital adaptation, digital transformation, scoping review

## Abstract

**Background:**

Patient organizations (POs) play a crucial role in supporting individuals with health conditions. Their activities range from counseling to support groups to advocacy. The COVID-19 pandemic and its related public health measures prompted rapid digital transformation efforts across multiple sectors, including health care.

**Objective:**

This study aimed to explore how POs digitally responded to pandemic-related circumstances, focusing on aspects such as the technologies used, positive outcomes, and challenges encountered.

**Methods:**

This scoping review followed the methodological guidance of the JBI (Joanna Briggs Institute) Scoping Review Methodology Group and adhered to the PRISMA-ScR (Preferred Reporting Items for Systematic Reviews and Meta-Analyses extension for Scoping Reviews) reporting guidelines. A systematic search of PubMed, the Web of Science Core Collection, and the WHO (World Health Organization) COVID-19 database, supplemented by a citation search approach, was conducted. The initial search was performed on November 10, 2022, and updated on November 8, 2023. Publications were eligible if they were published after November 30, 2019, and addressed pandemic-related digitalization efforts of POs, defined as nonprofit organizations with a focus on health-related support. A 2-step screening process was used to identify relevant literature. Data were extracted using a standardized table to capture aspects such as digital adaptation activities (eg, types of technologies implemented, positive outcomes, challenges, and facilitating factors) and coded inductively to identify similarities across included publications, and the findings were synthesized narratively.

**Results:**

The search and its subsequent update yielded 2212 records, with 13 articles included in this review. These articles revealed a range of PO services that were digitally adapted during the pandemic, with videoconferencing software emerging as the most commonly used tool (n=9 articles). The digital adaptation of group-based support activities was the most frequently reported transformation (n=9). Other adaptations included the digitalization of counseling services (n=3) and the delivery of information and education (n=3), including educational workshops, weekly webinars, and the dissemination of information through digital newsletters. While the use of digital formats, particularly for POs’ group activities, often increased accessibility by breaking down preexisting barriers (n=5), they also created new barriers for certain groups, such as those lacking digital skills or resources (n=4). Some participants experienced a loss of interpersonal aspects, like a sense of community (n=3). However, further findings suggest that the digital delivery of such group activities preserved essential interpersonal aspects (n=7) and a preference among some participants to continue digital group activities (n=4), suggesting the potential for sustainability of such options post the COVID-19 pandemic.

**Conclusions:**

The rapid digitalization efforts of POs demonstrate their adaptability and the potential of digital technologies to improve support services, despite some challenges. Future digitalization strategies should focus, among other things, on promoting digital literacy to ensure the accessibility and inclusiveness of digital services.

**Trial Registration:**

OSF Registries, https://osf.io/anvf4

## Introduction

Patient organizations (POs) provide crucial support to individuals affected by health conditions such as chronic diseases, and to their relatives. Support activities can range from direct services, such as individual counseling, support groups, and health education programs, to more indirect forms of support, such as advocacy in research or policy making [1-5]. In addition to differences in their primary purpose and scope of activities, POs also differ in organizational characteristics such as their internal structures and the financial resources available to them, adding to the complexity of the PO landscape. In Germany, for instance, Kofahl et al [3] found that almost half of the POs (47%) are run entirely by volunteers, while others have the capacity to employ full-time staff, ranging from 1 employee (17%) to 5 or more (15%). Membership sizes also vary widely, from a few individuals to more than 50,000 [3]. In the United States, Rose et al [4] found considerable financial variation among POs. While some highly professionalized POs reported annual revenues exceeding US $1 million, the median revenue for American organizations was US $299,140, suggesting that many operate on a smaller financial scale. This diversity among POs, in terms of their scope, structure, size, and financial resources, is also reflected in the lack of a universal definition, with terms such as “patient association,” “patient advocacy organization,” “self-help organization,” and “voluntary health agency” being used interchangeably [2-4]. To ensure readability and coherence in this review, we use “patient organization” as an umbrella term to encompass all such entities. Despite this broad terminology and considerable diversity, the majority of POs share 2 fundamental attributes. First, they mostly operate within a nonprofit framework, a characteristic frequently underscored in the definitions of these organizations [4,5]. It should be noted, however, that certain POs may have a legal structure that enables for-profit or hybrid models. Second, a unifying trait across this diverse spectrum is their commitment to supporting their respective target groups, underscoring the core mission that defines POs, as described before.

However, the onset of the COVID-19 pandemic drastically changed the circumstances of interpersonal interactions, often making it impossible to provide any kind of face-to-face service, as governments worldwide enacted public health protection measures that often included restrictions on movement and direct contact [6]. These policies triggered rapid digital transformations in various social domains, including work, education, business, and health care [7-9]. In health care, for example, several recent reviews have demonstrated a rapid significant increase in the use and adoption of telemedicine and eHealth apps [10-13].

Given the widespread impact of the pandemic and the resulting surge in the use of digital technologies, it is reasonable to assume that POs underwent similar digital adaptation efforts, changing their operational approaches and support delivery. The extent and effectiveness of these pandemic-induced digital adaptations likely varied across POs, reflecting not only the diversity described above but also varying levels of prepandemic digitalization. Before the pandemic, research indicated that the level of digitalization among POs varied widely. While many relied primarily on basic digital communication tools such as websites, email, and social media, others embraced advanced technologies by developing, for example, mobile apps that allow user interaction, web-based exercise programs, or electronic patient databases to enhance the use of digital patient data for research [14-16]. These differences in digitalization levels may be linked to the organizational characteristics described above, as well as additional factors such as the digital literacy of PO leadership and members and their demographic profiles [14].

However, despite the significant role of POs as supporters and advocates, we found no comprehensive reviews synthesizing the existing research literature on their digitalization efforts during the COVID-19 pandemic. While several reviews have addressed the use of digital technologies in health care during the pandemic [17-21], the specific focus on POs remains conspicuously absent. This gap leads us to our central research question, that is, how have POs adapted digitally in response to the COVID-19 pandemic?

To specifically address certain aspects of these activities, we pose the subquestions: (1) What digital technologies were adopted by POs in response to the pandemic? (2) What were the positive outcomes, challenges, and facilitating factors associated with these digital transformation efforts? Our findings on these matters may also eventually provide valuable insights for POs and similar organizations to guide future digitalization strategies and improve digital readiness for such unprecedented events, such as pandemics or situations where direct physical contact is restricted.

## Methods

### Methodological Framework

This scoping review is based on the methodological guidance developed by the JBI (Joanna Briggs Institute) Scoping Review Methodology Group [22]. For reporting, we adhered to PRISMA-ScR (Preferred Reporting Items for Systematic Reviews and Meta-Analyses extension for Scoping Reviews) guidelines [23]. The completed PRISMA-ScR checklist is provided in Multimedia Appendix 1.

### Protocol and Registration

A protocol was developed and registered with the Open Science Framework before the conduct of this review [24]. Given the iterative nature of scoping reviews, deviations from the initial protocol are generally possible [22,25]. All deviations are documented in the Multimedia Appendix 2 for transparency and justification.

### Eligibility Criteria

We established a set of specific criteria to systematically identify eligible articles among our search results (Table 1). To be included, an article had to meet all of the criteria, which we divided into 2 main types, formal aspects (criteria 1 to 5) and content-related aspects (criteria 6 and 7).

**Table 1:** Eligibility criteria.

Criterion category	Specific criterion
Formal	1. The full text of the article is available. 2. The article is published in English or German. 3. The article was published after November 30, 2019. 4. The article is not a duplicate publication. 5. All types of peer-reviewed articles^a^ published in a scientific journal, regardless of study design or methodological approach, are eligible.
Concept (PCC^b^ Component)	6. Eligible articles must report on digitalization efforts or initiatives within the PO^c^ that have been initiated, accelerated, or expanded to adapt to the COVID-19 pandemic, which may include the adoption, development, or use of any digital technology for PO activities or services.
Context (PCC Component)	7. Eligible articles must describe the efforts and initiatives of non-profit POs that provide direct or indirect support to individuals affected by health conditions^d^. Support groups will be included if they are affiliated with a PO. No geographic limitation was applied.

^a^In this review, study protocols and conference proceedings were not considered as full journal articles due to the preliminary or limited information they provide.

^b^PCC: Population, Concept, and Context.

^c^PO: patient organization.

^d^In this review, POs are defined as organizations separate from entities such as government agencies, faith-based groups, and academic institutions that may be non-profit and offer health-related support.

Regarding the latter, they correspond to the “Concept” and “Context” elements of our research question, derived from the Population, Concept, and Context (PCC) framework, which is often used to construct the title and main research questions of a scoping review [22,25] (Multimedia Appendix 3). Accordingly, articles were considered eligible if they addressed digitalization efforts initiated by POs during and in response to the COVID-19 pandemic. We defined digitalization efforts as the adoption, development, or use of digital technologies for activities previously conducted in person, or as the scaling up of existing digital services. To ensure a comprehensive overview, we considered the structural and functional variations of POs when defining our inclusion criteria. We defined POs by 2 main characteristics, first, their commitment to supporting individuals affected by specific health conditions. This may include direct support, like counseling or peer support, as well as indirect support, such as advocacy or research funding. Articles that addressed the digitalization of peer support groups were also included if such groups were associated with a PO. The second defining characteristic was their nonprofit nature, which is predominant in this sector.

We deliberately chose this broader definition, which aligns with the common characteristics outlined in the introduction, to account for the diverse international landscape of POs while ensuring a cohesive and comparable data set to analyze their digital responses to the pandemic. We intentionally excluded for-profit organizations, as their different operational and financial structures would complicate meaningful comparisons with nonprofits. This distinction ensures methodological consistency and precision in our analysis while recognizing the diversity of these organizations.

Regarding the former (formal inclusion criteria), these included article type, date, and language. We used these criteria to establish a consistent, transparent, and reproducible selection process. Acknowledging that the scope of published literature has not been previously summarized, we adopted a broad approach for certain criteria to ensure comprehensive coverage. For instance, we did not impose limitations on the type of article or study design.

### Information Sources and Search Strategy

To ensure that our systematic literature search was comprehensive, we selected the bibliographic databases PubMed, Web of Science Core Collection, and the WHO (World Health Organization) COVID-19 Research Database as sources of information. The search strategy was primarily developed by SW, who relies on specialized training and extensive experience in developing search strategies and conducting systematic searches gained through a previous professional position. It was first developed for PubMed and then adapted for the other databases. It consists of 3 thematic search blocks, each containing different synonymous free-text terms and, if available in the databases, corresponding controlled vocabulary terms, that consist of (1) patient organizations, (2) digitalization and digital technologies, and (3) the COVID-19 pandemic. The creation of the third search block was based on PubMed’s general COVID-19 article filter “LitCovid” [26]. Where possible, a publication language restriction was included in the search strings (refer to Multimedia Appendix 3 for the search strings). Quality assurance of the search strategy was performed by the other authors to ensure its robustness and comprehensiveness. The initial search was performed on November 10, 2022, and updated on November 8, 2023. Citavi 6 (Swiss Academic Software GmbH) was used for literature management. To further increase the comprehensiveness of our search and to identify additional eligible articles, a citation search was conducted.

### Selection Process

During the initial stage of our selection process, we used Citavi’s duplicate detection feature. Each potential duplicate was thoroughly reviewed by SW using criteria such as title, author, DOI, and journal. Only confirmed exact duplicates were removed, to ensure the integrity of the data set. To then systematically identify all relevant articles, we adopted a 2-step selection process. First, SW and CJ independently screened the titles and abstracts of all articles to determine eligibility based on our inclusion criteria (Table 1). When both reviewers considered an article potentially eligible, it was forwarded to full-text screening for further evaluation. Those that did not meet the eligibility criteria were excluded, with the first unmet criterion noted as the reason for exclusion. Conflicting assessments were discussed. If a consensus could not be reached, the article was also forwarded to full-text screening. Full texts of all preselected articles were then obtained and independently reviewed by SW and JL for final inclusion according to our inclusion criteria (Table 1). Conflicting assessments were discussed to reach a consensus. We did not need to involve a third independent reviewer in the selection process, as all conflicts were resolved at this stage. The screening process during the search update was also carried out using the 2-stage selection approach and was performed by SW and JL. We used Rayyan (Rayyan Systems Inc), a web-based software application designed to facilitate literature screening in systematic reviews, to conduct the selection process for both the initial and the update searches. The citation search was also performed independently by 2 reviewers (SW and CJ). It involved screening the reference lists of the included articles and searching Google Scholar for articles that cited them. The 2-step selection process was followed. The citation search approach was also applied to articles identified during the update of the systematic search, which was conducted by SW and JL.

### Quality Assessment

A critical appraisal of the methodological quality of the included articles was not performed. This is in line with the standard approach for scoping reviews to provide an overview of the existing sources of evidence on a particular topic, regardless of their methodological quality [25].

### Data Charting

To extract the relevant data from each included article, we developed a standardized table aligned with the PCC elements of our primary research question and subquestions. The extracted data included bibliographic information (eg, first author, title, and year of publication), methodological information (eg, study design and methodological approach), information on the POs (eg, type of entity, its specific health condition, and group represented), information on the digital adaptation activities (eg, types of digital technologies implemented, positive outcomes and facilitating factors identified, and challenges encountered).

Given the potential diversity in reporting styles, measures, and depth, and to maintain transparency and coherence, we focused our extraction on information explicitly linked to digital adaptation activities. This included aspects like the transformation of in-person services, members’ experiences with online support group activities, or the expansion of existing digital services. If a link to such efforts was not clearly identifiable, we excluded that information from the data extraction. For example, a study reporting a lack of funding as a major challenge for POs during the pandemic was not included unless the challenge was explicitly related to funding for such digitalization measures. To ensure consistency and accuracy, SW and JL collaboratively extracted the data, with each handling half of the included articles. The data was initially extracted verbatim so as not to miss important details. This approach included a range of data, from concise statements to entire paragraphs. Subsequently, the extracted information was condensed to distill the main points and to facilitate focused analysis.

### Analysis and Data Synthesis

After condensing the textual data, we proceeded with inductive coding. Thematic codes were assigned to the condensed data to reflect the primary information reported. These codes were then organized into broader categories. Whenever possible, these codes, as well as the corresponding broader categories, were applied across the data of all included articles, to allow for a structured and comprehensive analysis.

In the narrative synthesis of the data, which forms the core of our results section, we aimed to highlight similarities and differences among the included articles and to describe potential trends using a slightly quantitative approach by presenting the frequency of reporting (eg, regarding the digital technologies used or the services that had been digitally transformed).

To achieve this, we first summarized the characteristics of the included articles, followed by information on the POs highlighting key aspects and variations. The reported characteristics of the digital adaptation efforts were then synthesized, with a focus on identifying and describing common trends and approaches.

## Results

### Search and Selection of Articles

**Figure 1:**
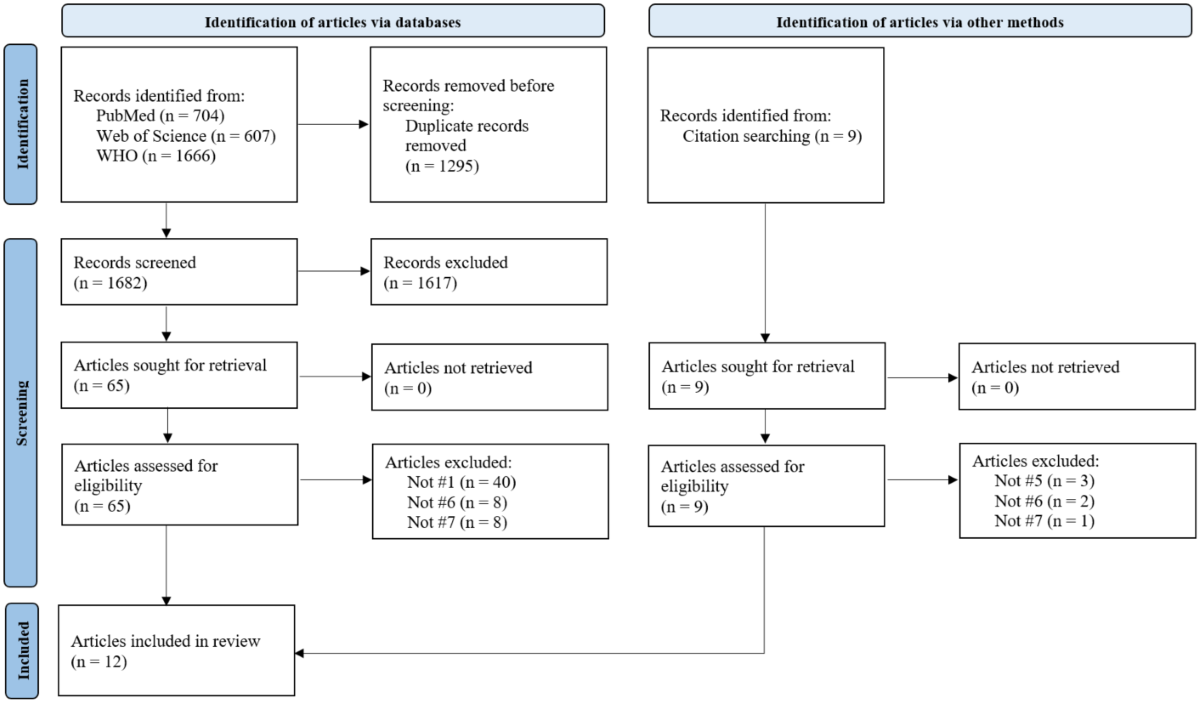
PRISMA (Preferred Reporting Items for Systematic Reviews and Meta-Analyses) flow diagram of the selection process during the initial search. WHO: World Health Organization.

**Figure 2:**
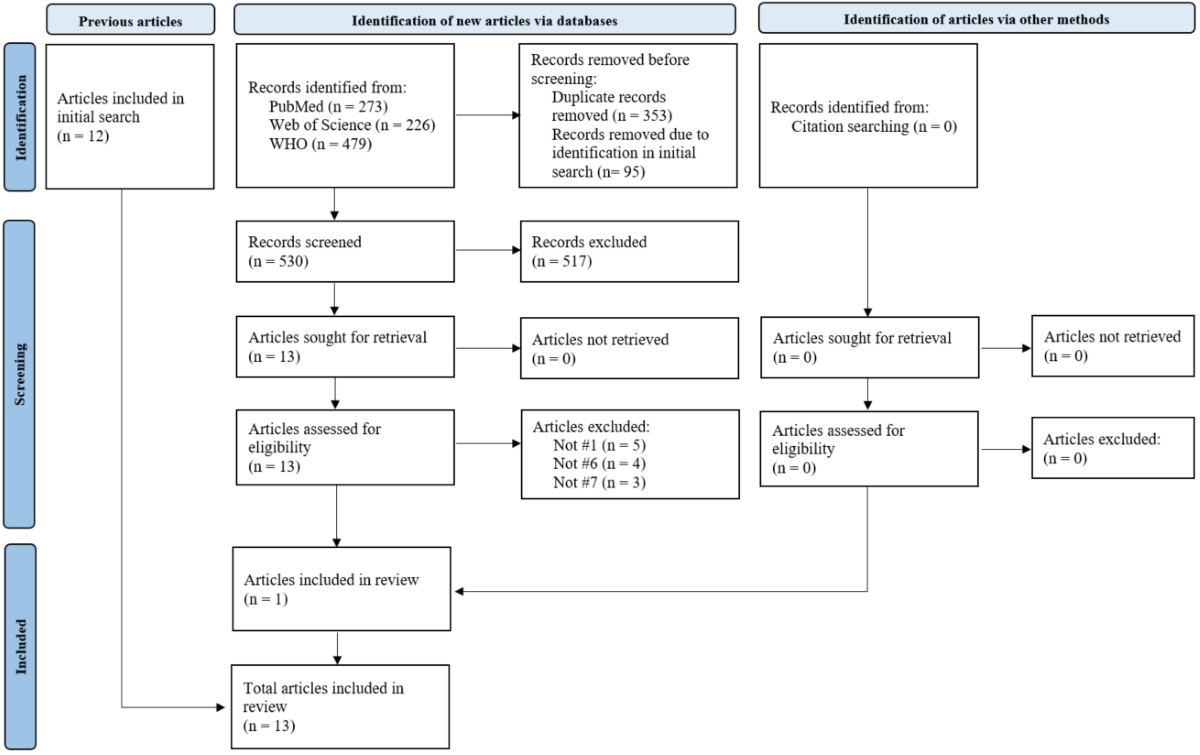
PRISMA (Preferred Reporting Items for Systematic Reviews and Meta-Analyses) flow diagram of the selection process during the update search. WHO: World Health Organization.

The systematic search of bibliographic databases, along with its update, yielded a total of 2212 results after the removal of duplicates. Of these, 10 references met the eligibility criteria and were included in our review. The citation search yielded 3 additional eligible references. Thus, after the completion of all screening steps, 13 articles were finally included. Figure 1 displays the selection process during the initial search using a PRISMA (Preferred Reporting Items for Systematic Reviews and Meta-Analyses) flow diagram [27]. The search update is shown in Figure 2.

### Characteristics of Included Articles

Of the 13 included references, 9 were original research articles. Of these, most were empirical studies employing a qualitative (n=4) or mixed methods approach (n=4). Of the remaining 4 articles, 1 was based on a survey but was published as a data note due to its unexpectedly small sample size [28]. The other 3 articles can be broadly categorized as experience reports, as they aimed to describe practical examples or lessons learned regarding digital transformation, without collecting data in this context [29-31].

Most of the included articles were published during the first 2 years of the COVID-19 pandemic. Of the 3 included articles published in 2023, Bouey et al [32] was initially identified as a preprint, flagged as relevant, and included when it was fully published. Constantini et al [33] was identified through the search update.

The majority of the included articles (n=8) reported receiving financial support from a PO or indicated varying degrees of involvement by representatives of a PO [29-32,34-36], or an umbrella organization of POs [37]. Table 2 presents the objectives of the included articles, while Table 3 outlines their general characteristics. Also refer to Multimedia Appendix 4.

**Table 2:** Objectives of included articles (N=13).

Reference	Objective
Beck et al [38]	Evaluate SMART^a^ (Self-Management and Recovery Training) Recovery Australia’s scale-up of online support groups during the COVID-19 pandemic.
Bergmans et al [29]	Present lessons learned from transitioning the Skills for Safer Living intervention to a digital format.
Bouey et al [32]	Explore the challenges Chinese POs^b^ faced during the first COVID-19 lockdown and how they responded.
Chung et al [37]	Examine the impact of the pandemic on rare disease POs in the Asia-Pacific region.
Constantini et al [33]	Compare the experiences of members of support groups that have transitioned to a digital format with general findings from the literature on online support groups.
Kelly et al [30]	Discuss the global impact of COVID-19 on SMART Recovery International with facilitators from different regions.
Lamont et al [36]	Explore stroke survivors’ perceptions of social support and shared identity within stroke groups during COVID-19 and its impact on psychosocial health.
Marks et al [35]	Investigate the effectiveness and experiences of participants in online tinnitus support groups and educational webinars implemented during COVID-19.
McMullan et al [28]	Examine the impact of the pandemic on rare disease POs in Ireland and the United Kingdom.
Nemeth Blažić et al [31]	Describe, among other things, the digitalization of a PO’s voluntary counseling and testing services for HIV, hepatitis C, and other sexually transmitted infections during the pandemic.
Penfold and Ogden [39]	Explore Gamblers Anonymous members’ experiences with digitally delivered group meetings during the pandemic to understand the effectiveness and support provided compared to in-person meetings.
Seckam and Hallingberg [34]	Examine stroke survivors’ experiences with the transition from in-person to digital choir sessions during COVID-19.
Senreich et al [40]	Explore 12-step program attendees’ experiences with in-person group meetings transitioning to digital formats.

^a^SMART: Self-Management and Recovery Training.

^b^PO: patient organization.

**Table 3:** Characteristics of included articles (N=13).

Characteristics		Articles, n (%)	References
**Publication year^a^**			
	2021	5 (38)	[28-30,34,37]
	2022	5 (38)	[31,35,36,39,40]
	2023	3 (23)	[32,33,38]
**Article type**			
	Data note	1 (8)	[28]
	Report	3 (23)	[29-31]
	Research article	9 (69)	[32-40]
**Methodological approach**			
	Mixed-method	4 (31)	[32,33,37,38]
	Nonempirical	3 (23)	[29-31]
	Qualitative	4 (31)	[34,35,39,40]
	Quantitative	2 (15)	[28,36]
**PO^b^ involvement^c^**			
	Funding by PO	1 (8)	[36]
	Involvement of PO representatives	5 (38)	[29-32,34]
	Involvement of representatives from an umbrella organization of POs	1 (8)	[37]
	PO representative involvement and PO funding	1 (8)	[35]

^a^Percentages in the "Publication year" category do not exactly total 100 due to rounding.

^b^POs: patient organizations.

^c^The category "PO involvement" does not contain all included publications, as not every article reported a form of involvement with a patient organization.

### Characteristics of Patient Organizations

#### Overview

The majority of the included articles (n=8) addressed POs involved in a broad range of activities, including direct support services, advocacy, or research participation [28,29,31,32,34-37]. Meanwhile, 5 articles reported on POs, such as SMART Recovery (Self-Management and Recovery Training), that primarily provide peer support groups [30,33,38-40]. Of all included articles, 8 specifically explored the digital adaptation of POs’ group-based support activities without addressing other organizational elements [29,30,33,34,36,38-40]. Moreover, a focus on well-established POs such as SMART Recovery [30,38], the Stroke Association [34,36], and the Canadian Mental Health Association [29] was predominant among the included articles.

The majority of included articles focused on POs within specific countries. Most articles (n=8) reported on individual POs operating within a single country [29,31,33-36,38,39], while 2 explored multiple POs within the same national context [32,40]. In addition, 2 articles expanded their scope to include POs in a transnational region [28,37], and another provided an international overview by focusing on SMART Recovery International and its network of global affiliates [30]. Among all the articles, POs based in the United Kingdom were the most commonly reported on (n=5).

In terms of the health conditions addressed by the POs, addictive behaviors were the most common (n=4), followed by stroke (n=2), infections with HIV, AIDS or other sexually transmitted infections (n=2), and rare diseases (n=2). Table 4 provides an overview of aspects related to the PO settings, as reported in the included articles. Also refer to Multimedia Appendix 4.

**Table 4:** Characteristics of Patient organizations as reported in the included articles (N=13)^a^.

Characteristics			Articles, n (%)		References
**Indication**					
	Addictive behaviors	4 (31)		[30,38-40]	
	Dementia	1 (8)		[33]	
	HIV, AIDS, and other sexually transmitted infections^b^	2 (15)		[31,32]	
	Mental health	1 (8)		[29]	
	Rare diseases	2 (15)		[28,37]	
	Stroke	2 (15)		[34,36]	
	Tinnitus	1 (8)		[35]	
**Geographic location**					
	Australia	2 (15)		[37,38]	
	Austria	1 (8)		[33]	
	Canada	1 (8)		[29]	
	China	2 (15)		[32,37]	
	Croatia	1 (8)		[31]	
	Hong Kong	1 (8)		[37]	
	India	1 (8)		[37]	
	Ireland	1 (8)		[28]	
	Japan	1 (8)		[37]	
	Malaysia	1 (8)		[37]	
	New Zealand	1 (8)		[37]	
	Philippines	1 (8)		[37]	
	Singapore	1 (8)		[37]	
	Taiwan	1 (8)		[37]	
	Transnational (PO^b^ with affiliations in Australia, Brazil, Denmark, Hong Kong, Ireland, Malaysia, Spain, United Kingdom, and United States)	1 (8)		[30]	
	United Kingdom	5 (38)		[28,34-36,39]	
	United States	1 (8)		[40]	
**Digitally adapted PO^c^ activities and services during the COVID-19 pandemic**					
	Group-based support activities	9 (69)		[29,30,33-36,38-40]	
	Communication and counseling	4 (31)		[28,31,32,37]	
	Information provision and educational activities	3 (23)		[28,35,37]	
	Operational activities	2 (15)		[28,37]	

^c^Articles may appear in multiple categories within this table, as some studies report on multiple geographic locations or digitally adapted activities/services. Percentages are calculated based on the total number of 13 articles.

^b^SMART Recovery International.

^c^PO: patient organization.

#### Patient Organizations’ Digital Responses to the COVID-19 Pandemic

**Figure 3:**
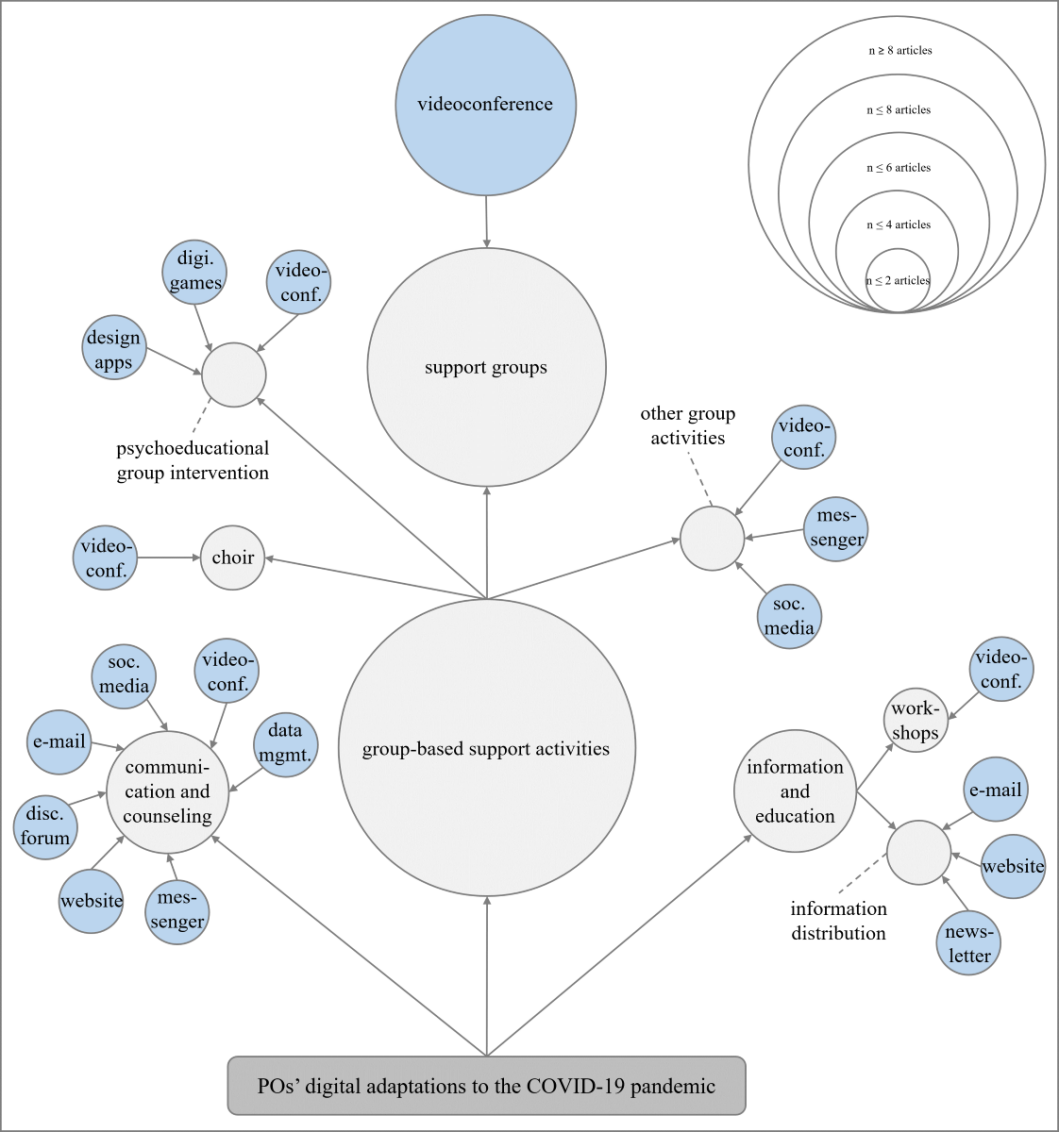
Patient organizations’ activities and services were digitally adapted during the COVID-19 pandemic (gray bubbles) and the technologies used (blue bubbles). PO: patient organization.

Among the articles reviewed, digital adaptations of various PO activities in response to COVID–19–related public health measures were described. Most (n=9) focused on the digitalization of a specific PO activity [29-31,33,34,36,38-40], while 4 explored the digital transformation of multiple activities [28,32,35,37]. These digitally adapted services and activities can be broadly categorized into 3 main areas, that are group-based support activities, communication and counseling, as well as information provision and educational activities (refer to Figure 3).

### Group-Based Support Activities

#### Overview

The digitalization of group-based support activities is the most frequently reported aspect (n=9), with peer support groups discussed in 7 articles [30,33,35,36,38-40]. This includes both the transformation from face-to-face to online support groups [33,35,39,40], as well as the significant expansion of existing online support groups that were previously less common and mainly held in conjunction with face-to-face meetings [34]. Kelly et al [30] discussed the response of SMART Recovery International, a global community of peer support groups for addictive behaviors. In some countries, like the United States, this involved notably increasing the number of online support groups to compensate for the discontinuation of in-person groups, while in others, such as Denmark, digital options were introduced for the first time. Lamont et al [36] described various digital communication methods used by facilitators and members of stroke groups of the Stroke Association (United Kingdom) to maintain engagement. Originally, peer support, creative arts, and stroke cafés were among the activities of these groups. However, the extent of digital transition remains ambiguous, as the original core activities were not necessarily directly adapted, with Lamont et al [36] reporting that digital tools were primarily used to keep in touch. Other POs’ group activities that were digitally transformed during the pandemic include a choir [34] and a psychoeducational group intervention [29].

In terms of digital technologies, videoconferencing, including platforms such as Zoom, was mentioned in 8 articles, making it the most commonly used tool in the process of digital adaptation [29,33-36,38-40]. Kelly et al [30] did not specify the technology used to deliver support groups digitally. Refer to Table S1 in Multimedia Appendix 5, for further details.

#### Positive Outcomes

The most commonly reported benefits of digitally adapting group-based support activities during the pandemic were preserving interpersonal dynamics and increasing accessibility (refer to Table S2 in Multimedia Appendix 5, for details on other positive outcomes).

#### Interpersonal Dynamics

Of the 9 articles addressing group-based support activities, 7 reported that the interpersonal nature of such activities was successfully preserved in the digital setting [29,33-36,38,39]. For instance, Seckam and Hallingberg [34] observed that participants felt inspired by the meetings with their fellow members and experienced a sense of belonging in the digital environment, which helped reduce feelings of social isolation. Similarly, Marks et al [35] reported that participants felt less isolated, experienced social or emotional connections with other group members, and shared information and stories within the digital environment. Penfold and Ogden [39] also observed social comparison as well as interpersonal dynamics such as social affirmation, solidarity, and feelings of togetherness in digitally delivered group meetings. Both conclude that key elements of group meetings were preserved during the digital transition [35,39]. Lamont et al [36] observed a high level of social identification of members with their group during the pandemic, resulting in more positive psychosocial outcomes. The authors attributed this to groups maintaining collective and individual interactions, primarily through communication tools such as email, video calls, and text messages.

#### Accessibility

Several articles (n=5) referred to the impact of the digital transition on the accessibility of group-based support activities. For instance, 3 articles reported improved access for people who were previously unable to participate in person due to barriers related to physical or mental health conditions, work, or financial resources [29,35,40]. By moving to digital formats, some POs also expanded the accessibility of their group activities nationally or even internationally [30,35,39,40]. This newly gained accessibility was viewed positively by some participants. Penfold and Ogden [39] highlighted that participants appreciated being able to choose from a wider range of meetings, and Senreich et al [40] observed that the increased diversity of participants, due to easier access, was positively perceived.

#### Challenges and Barriers

At the same time, this shift to digital formats introduced several challenges to the delivery of group-based support activities. Access barriers, particularly for those individuals lacking digital resources or skills, were the most commonly reported [34-36,40]. Seckam and Hallingberg [34] explicitly discussed these as an ethical challenge, highlighting the unintentional exclusion of some members from virtual choral activities due to these factors. Furthermore, the accessibility of group activities was sometimes negatively impacted by technological difficulties, such as poor internet connections [33,38].

Challenges related to privacy and anonymity were reported in three articles, including difficulties in managing privacy in the digital setting due to the facilitator’s limited control [29], group member concerns about maintaining anonymity in group meetings [39], and incidents of “zoom bombing” with disruptive behavior [40].

In contrast to the finding that maintaining the dynamics of group interactions was identified as a benefit of digital formats, 3 articles reported that some group members were less satisfied with such formats, particularly in terms of interpersonal aspects, such as feeling rather disconnected from the group and lacking a sense of community [35,39,40]. Furthermore, the absence of informal conversations and interactions typically surrounding in-person meetings was viewed negatively [40], posing challenges in developing relationships within the groups for some [35]. For more details, refer to Table S3 in Multimedia Appendix 5.

#### Facilitating Factors

From the perspective of an internationally-operating PO, Kelly et al [30] reported on the challenges SMART Recovery International faced in digitally transforming support groups in countries where such digital options were not previously established. Here, the digital environment had to be developed from the ground up and facilitators had to be trained. However, the authors also mentioned that collaborating transnationally and sharing experiences with SMART Recovery affiliates in countries with preexisting online support groups proved to be beneficial. Participants’ previous experience with digital technologies [33,34], the presence of experienced group facilitators [33,35], and government funding [30,38] also had a positive impact on the digital transformation of group activities. Refer to Table S4 in Multimedia Appendix 5, for more details.

### Communication and Counseling

Chung et al [37] and McMullan et al [28], based on their surveys with representatives of rare disease POs, reported that the majority of participating organizations used digital communication tools to maintain contact with their members and provide ongoing support during the pandemic. This included enabling interactions through video calls, social media, and discussion forums.

In addition, Chung et al [37] described the digitalization of counseling services that were previously offered in person. Bouey et al [32] and Nemeth Blažić et al [31] also discussed this transition, focusing on POs that provide support and care for individuals with sexually transmitted infections, particularly HIV infection and AIDS, where counseling is an integral part of their prevention and testing activities. Various digital technologies were used to maintain support through counseling during the pandemic, such as videoconferencing software, email, and social media.

Chung et al [37] reported that relying on digital communication tools during the pandemic posed challenges to adequately supporting all members, as not all had access to these tools or were skilled enough to use them.

We could not identify any information on facilitating factors specifically associated with the digital transformation of POs’ communication and counseling services (Table S1-S4 in Multimedia Appendix 6).

### Information Provision and Educational Activities

The digital adaptation of POs’ information provision and educational activities was addressed in 3 of the included articles [28,35,37]. Marks et al [35] described the digital delivery of educational workshops through Zoom, while McMullan et al [28] briefly mentioned the implementation of weekly webinars and the distribution of information updates and newsletters. Similarly, Chung et al [37] reported the use of newsletters, along with the use of POs’ websites, as methods of disseminating information to members during the pandemic.

Marks et al [35] observed that participants in the educational workshops delivered via Zoom experienced social support and a sense of collectivity, echoing findings from digital group-based support activities. They also deduced from their qualitative interview data that professional facilitation and moderation created a positive learning environment where participants felt motivated and comfortable to engage. Attributes specific to the digital format, such as the ease of information sharing through chat, enhanced the learning experience. However, the authors also described challenges associated with the use of digital technology for some participants. Further details are provided in Table S1-S4 in Multimedia Appendix 6.

### Operational Activities

Chung et al [37] and McMullan et al [28] also addressed how POs have digitally transformed their operation. McMullan et al [28] specifically mentioned the transition of committee meetings to videoconferencing formats. Chung et al [37] also noted the digitalization of meetings, though they did not specify the types, and further addressed the broader digitalization of organizational operations as an adaptive response to the pandemic. Their survey of directors and representatives of rare disease POs in the Asia-Pacific region revealed that the “digitalization of operation” was most frequently stated as the predominant factor contributing to the perceived success of POs during the pandemic. However, the survey also showed that the actual success of these digital adjustment efforts varied by geographic region. For example, all 15 Australian organizations reported successful digital adaptation of their operations, while none of the 18 participating POs from Hong Kong were able to digitally adapt. The authors hypothesize that this disparity may be due to differences in the availability of the necessary digital infrastructure. Refer to Tables S1-S4 in Multimedia Appendix 6, for more details.

## Discussion

### Scope and Context

Based on the results of our systematic literature search, this review provides a comprehensive overview of how various POs adapted their services and operations to digital formats in response to pandemic-related circumstances. It covers a wide range of organizational types, health-related indications, and geographic areas, highlighting the broad impact of the pandemic.

### Principal Findings on Patient Organizations’ Digital Responses

Our analysis reveals that the COVID-19 pandemic prompted various digital responses from POs. It led to the adoption of new digital solutions or, in some cases, the significant expansion of existing digital services, to maintain essential activities under pandemic conditions and to meet support needs. All articles reported largely successful digital efforts by POs and their associated support groups, ensuring the continuation of their services in the midst of the pandemic. This is consistent with broader trends observed in the health care sector, where rapid digital transitions during the pandemic played a critical role in maintaining service delivery [13,17,18,20,21].

As all included articles focus on the immediate impact of the pandemic and the rapid adoption of digital solutions, it is not possible to reach definitive conclusions about the long-term impact of the pandemic on digitalization within these organizations, or its sustainability. However, our findings from 4 articles suggest a preference among some participants for the continuation of digital group activities [33,35,38,40]. This observation suggests that digital options might beneficially be preserved alongside the resumption of in-person meetings, particularly where digital experiences have been positive. Therefore, future research could explore the long-term implications of pandemic-induced digital transformations within these POs.

Another notable finding is that the digital transformation of group-based support activities is the most frequently and extensively reported response in our sample (n=9). This trend may be related to the fact that providing peer support, such as through support groups, is a core task of many POs [2,4,5]. The pressing need to digitize these services during the pandemic, which was likely a priority for organizations to continue providing support, may explain the extensive reporting of this transition.

### Digital Technologies

The predominant use of videoconferencing software during the pandemic-driven digitalization is consistent with trends observed in various sectors, such as higher education [41] and health care [17]. This widespread adoption may be related to the nature of these tools, which enable audiovisual communication and are therefore closest to face-to-face interaction. Consequently, videoconferencing offers a more comprehensive mode of interaction, potentially making it a more appealing alternative for social interaction than purely speech- or text-based forms. Before conducting our review, we had considered the possibility that POs might develop specific digital solutions, most likely in collaboration with software developers, to better customize their services and meet the needs of their members. However, our findings did not indicate such developments. This may be due to the rapid and urgent nature of the pandemic-related digital adaptations, which likely made the development of specialized technologies impractical at the time. In addition, the relatively high cost may have been a limiting factor or existing tools may have been sufficient for the needs of POs.

### Positive Outcomes, Challenges, and Facilitating Factors

The majority of articles addressing group activities reported that participants’ interpersonal experiences in online support groups were similar to those in face-to-face groups, including a sense of togetherness. This observation aligns with similar trends reported in studies on various digital support group formats that emerged independently of the COVID-19 pandemic [42-44]. Consequently, such groups may indeed serve as adequate alternatives to face-to-face group activities of POs. Another positive outcome highlighted in several articles, primarily regarding the digitalization of group activities, is increased accessibility. This shift has effectively removed several former barriers, allowing participants to access meetings from anywhere. This has been particularly beneficial for individuals with financial or mobility constraints. This reported benefit of improved access through digital technologies is consistent with previous findings, both during [13,20] and outside the pandemic period [42]. However, our review also shows that the shift to digital formats has simultaneously created new barriers, particularly for those with limited digital literacy or resources. These challenges also appear to transcend the pandemic context, as evidenced by other findings on digital health in both pandemic [13,20] and non-pandemic settings [45,46]. This dichotomy points to the dual nature of digital formats, as they can both bridge and widen access gaps and underscores the importance of nuanced consideration.

Regardless of the specific service or activity, we found that comparatively few factors were consistently reported as facilitators of digital transformation. The beneficial impact of skilled facilitators on the smooth digital adaptation of support groups and educational workshops, as reported by Marks et al [35] and Constantini et al [33], is also highlighted in a review not specifically related to the pandemic [46], making this a potentially relevant general recommendation for the future delivery of digital activities. From a broader perspective not focused on group activities, Chung et al [37] identified preexisting digital resources and experience in digitally delivering such services and activities as important facilitators of digital adaptation.

In conclusion, when establishing any kind of digital service within POs, it is likely to be advantageous to promote digital literacy among members and participants and to create solutions for those without access to digital technologies, as well as to be digitally equipped and experienced as an organization.

### Ethical Issues

In contexts such as digital transformation in health care, ethical implications such as privacy and justice, as well as related issues, are frequently discussed [47]. Notably, as mentioned above, only Seckam and Hallingberg [34] reported ethical considerations related to the limited accessibility of digital formats and the unintentional exclusion of some group members due to digital literacy gaps or a lack of necessary technology. While other articles [34-36,40] also reported accessibility challenges, they did not explicitly categorize them as ethical issues. Bergmans et al [29], Penfold and Ogden [39], and Senreich et al [40] observed privacy concerns, although not explicitly framed as ethical issues either. These challenges regarding fair access and privacy align with those identified in the broader digital health landscape, where ethical guidance is suggested as an initial step to address such concerns [47]. However, only Bergmans et al [29] reported mitigating actions, such as “coaching” sessions to help participants navigate the privacy settings of digital platforms. Other articles did not specify the approaches taken by POs to address these potential ethical challenges.

### Limitations of Findings

First, our analysis is based on a limited sample of publications that may not fully represent the situation of all POs. Our search primarily yielded articles that focused on organizations in Europe, particularly the United Kingdom. Therefore, our findings may not fully capture the global situation. It is possible that digital responses related to the pandemic may have varied significantly in countries not included in our sample. In addition, the majority of the articles focused on well-established organizations with the resources to actively support research. Of these, 7 articles received funding from or were developed in an active collaboration with POs, suggesting that these factors may have influenced the scope of the available research on this topic. As a result, the experiences and perspectives of smaller, less financially equipped organizations may be underrepresented in our findings, despite representing a large proportion of POs in countries, such as Germany or the United States [3,4]. In conclusion, our findings indicate that the research literature predominantly focuses on more professionalized POs and entities in specific geographical regions. Hence, a need for more diverse research is apparent. Further, we found that despite the diverse activities of POs, the articles in this review did not address instances in which these organizations acted as advocates, which may include efforts to influence health policy making, collaborate with other health care stakeholders, or engage in research. Consequently, it remains unclear whether these specific activities were also adapted to digital formats, suggesting potential areas for further research.

Having acknowledged these limitations, it is important to clarify our objective. The primary purpose of this review was not to generate generalizable findings or to provide a comprehensive picture of the global situation. Rather, our aim was to determine what literature has been published on the topic, describe individual findings, identify emerging trends, and highlight research gaps. In fact, the absence of articles addressing certain gaps identified in our review is more indicative of a lack of diversity in the published literature. This may be due to factors such as POs lacking the necessary financial or human resources for research involvement, or possibly because engaging in research or publishing in academic journals is not a priority for some POs.

Finally, the quality of reporting within our sample varies, particularly in terms of depth. For example, most articles did not focus on the digital transformation process as such. This variation means that the amount of information we gathered and extracted varied. As a result, not all aspects of digital transformation were reported in each article, nor were they necessarily covered to the same extent.

### Methodological Limitations

A challenge in developing our search strategy was the lack of consistent terminology and definitions for POs. Hence, we refined our search strategy, incorporating various synonyms to retrieve relevant literature. After the initial search and screening, we identified additional terms as potentially relevant, such as “voluntary health agency.” The fact that adding these terms to the search strategy did not yield any additional relevant publications gives us further confidence that our search was sufficiently sensitive.

Also, we used a definition of POs that may reflect a rather Western perspective. Potentially, even broader inclusion criteria might have yielded more publications. However, this approach may have resulted in a sample with significant variation across organizations, making comparisons impossible. In addition, by limiting our review to nonprofit POs, the scope of our findings may be somewhat narrowed, as POs with different operational modes are not represented. Nevertheless, this approach reflects the predominant structure within the sector.

Finally, we included only articles published in English or German to ensure feasibility. The overall search results in PubMed, for example, showed only a modest increase in search results (0.87%) when no language filter was applied, and hence this language restriction appears to have been negligible.

### Conclusion

Our findings on these rapid transitions can be taken as an indicator of the resilience and adaptability of these POs, and underscore the significant potential of digital technologies to enhance support services in such unprecedented times.

Based on the various aspects we identified in this review, potentially relevant recommendations for future PO digitalization strategies relate to promoting digital literacy among members and participants, creating solutions for those without access to digital technologies or those who may not wish to use them, and training PO staff to provide skilled and supportive delivery of digital services.

Our findings also highlight several research gaps. For example, there seems to be a predominant focus in the literature on well-established organizations in Western countries. This may overlook the unique experiences of smaller, less well-funded POs or those in different geographical areas. In addition, the current literature does not provide insights into areas such as advocacy or research engagement and their digital adaptations, suggesting potential areas for further research. Furthermore, while our review highlights the immediate digital responses of POs to the pandemic, the long-term sustainability and impact of these adjustments remain unclear. Future research should explore these aspects to fully understand the long-term impact of the COVID-19 pandemic on POs.

## Appendix

Multimedia Appendix 1 PRISMA-ScR (Preferred Reporting Items for Systematic Reviews and Meta-Analyses extension for Scoping Reviews) checklist.

Multimedia Appendix 2 Deviations from protocol.

Multimedia Appendix 3 PCC (Population, Concept, and Context) mnemonic and search strategies.

Multimedia Appendix 4 Characteristics of included articles and patient organizations.

Multimedia Appendix 5 Consolidated findings on patient organizations’ digital adaptations of group-based support activities.

Multimedia Appendix 6 Consolidated findings on patient organizations’ digital adaptations of communication, counseling, information provision, educational activities, and other operational aspects.

## Abbreviations

JBI: Joanna Briggs Institute
PCC: Population, Concept, and Context
PO: patient organization
PRISMA: Preferred Reporting Items for Systematic Reviews and Meta-Analyses
PRISMA-ScR: Preferred Reporting Items for Systematic Reviews and Meta-Analyses extension for Scoping Reviews
SMART: Self-Management and Recovery Training
WHO: World Health Organization
