# Supplementary material for: Patient Organizations’ Digital Responses to the COVID-19 Pandemic: Scoping Review
Source: J Med Internet Res. 2024 Dec 20;26:e58566. doi: 10.2196/58566 (PMC11699494; doi:10.2196/58566)
Supplement: Multimedia Appendix 2 [file jmir_v26i1e58566_app2.pdf]

Multimedia Appendix 2: Deviations from Protocol

Table S1. Comprehensive list of deviations from the protocol.

1	The main research question was further refined prior to conducting the systematic search to emphasize the goal of this review to provide insight into how patient organizations (POs) digitally responded to pandemic-related circumstances. The original draft, as presented in our protocol, is worded more broadly, suggesting the exploration of the overall impact of the pandemic on the digitalization within POs. Such an overall impact might be more of a long-term effect that should be addressed in a future review with the appropriate temporal distance to the pandemic.
2	Sub-questions were further refined prior to the systematic search without changing their initial meaning.
3	The wording of eligibility criterion #6 was further refined prior to the systematic search without changing its initial meaning.
4	The wording of eligibility criterion #7 was further refined prior to the systematic search without changing its initial meaning. It was also decided to exclude COVID-19-specific online support groups from the review to maintain thematic consistency. The focus is on the adaptive responses of established patient organizations and support groups, rather than services that are newly established in response to COVID-19-specific needs, such as groups for long COVID patients. It was recognized that COVID-19-specific services represent a distinct area of research that may need to be addressed in other reviews.
