# Supplementary material for: Patient Organizations’ Digital Responses to the COVID-19 Pandemic: Scoping Review
Source: J Med Internet Res. 2024 Dec 20;26:e58566. doi: 10.2196/58566 (PMC11699494; doi:10.2196/58566)
Supplement: Multimedia Appendix 3 [file jmir_v26i1e58566_app3.pdf]

Multimedia Appendix 3: PCC Mnemonic and Search Strategies

Table S1. PCC mnemonic.

**P**	**Population:**<br>N/A[1]
**C**	**Concept:**<br>Digitalization efforts and initiatives implemented to adapt to the COVID-19 pandemic.
**C**	**Context:**<br>Patient organizations as well as associated peer support groups.

Table S2. Search strategy for PubMed.

Search	Query
#1	Self-Help Groups[mh] or Patient Advocacy[mh]
#2	Organizations, Nonprofit[mh:noexp]
#3	#2 and (health[tiab] or disease[tiab] or patient*[tiab] or advoca*[tiab] or "self-help*"[tiab] or selfhelp*[tiab] or support*[tiab])
#4	"self-help*"[tiab] or selfhelp*[tiab] or "mutual aid"[tiab] or "mutual help*"[tiab] or "support group*"[tiab] or "peer support"[tiab] or "mutual support"[tiab] or "group support"[tiab] or "patient advoca*"[tiab] or "patient represent*"[tiab] or "patient organization*"[tiab] or "patient organisation*"[tiab] or "patient support organization*"[tiab] or "patient support organisation*"[tiab] or "patient interest group*"[tiab] or "patient association*"[tiab] or "patient support association*"[tiab] or "health advoca*"[tiab] or "health interest group*"[tiab] or "rare disease organization*"[tiab] or "rare disease organisation*"[tiab]
#5	"non-profit organization*"[tiab] or "non-profit organisation*"[tiab] or "nonprofit organization*"[tiab] or "nonprofit organisation*"[tiab] or npo[tiab] or npos[tiab] or "not-for-profit organization*"[tiab] or "not-for-profit organisation*"[tiab] or nfp[tiab] or nfps[tiab] or "non-government organization*"[tiab] or "non-government organisation*"[tiab] or "nongovernment organization*"[tiab] or "nongovernment organisation*"[tiab] or "non-governmental organization*"[tiab] or "non-governmental organisation*"[tiab] or "nongovernmental organization*"[tiab] or "nongovernmental organisation*"[tiab] or ngo[tiab] or ngos[tiab]

[1] No specific health condition or population was addressed in this scoping review.

Search	Query
#6	#5 and (health[tiab] or disease[tiab] or patient*[tiab] or advoca*[tiab] or "self-help*"[tiab] or selfhelp*[tiab] or support*[tiab])
#7	#1 or #3 or #4 or #6
#8	Digital Technology[mh] or Telecommunications[mh:noexp] or Telemedicine[mh:noexp] or Internet[mh:noexp] or Internet Use[mh] or Internet-Based Intervention[mh] or Web Browser[mh] or Software[mh:noexp] or Computers, Handheld[mh] or Cell Phone[mh] or Cell Phone Use[mh] or Mobile Applications[mh] or Wearable Electronic Devices[mh] or Electronic Mail[mh] or Videoconferencing[mh] or Social Media[mh] or Online Social Networking[mh] or Blogging[mh] or Multimedia[mh]
#9	digital*[tiab] or digitiz*[tiab] or digitis*[tiab] or online[tiab] or "on-line"[tiab] or webbased[tiab] or "web-based"[tiab] or virtual*[tiab] or cyber[tiab] or computerized[tiab] or computerised[tiab] or technological*[tiab] or electronic*[tiab] or "e-health"[tiab] or ehealth[tiab] or "mobile health"[tiab] or "m-health"[tiab] or mhealth[tiab] or telemedicine[tiab] or "tele-medicine"[tiab] or telehealth[tiab] or "tele-health"[tiab] or internet[tiab] or web[tiab] or software[tiab] or "cell phone*"[tiab] or "cellular phone*"[tiab] or "mobile phone*"[tiab] or smartphone*[tiab] or "smart phone*"[tiab] or "smart device*"[tiab] or tablet*[tiab] or app[tiab] or apps[tiab] or application*[tiab] or "wearable device*"[tiab] or "e-mail*"[tiab] or email*[tiab] or videoconferenc*[tiab] or "video-conferenc*"[tiab] or chat*[tiab] or "discussion forum*"[tiab] or "discussion fora"[tiab] or "social media"[tiab] or facebook[tiab] or youtube[tiab] or whatsapp[tiab] or instagram[tiab] or tiktok[tiab] or twitter[tiab] or blog*[tiab] or webinar*[tiab] or podcast*[tiab] or webcast*[tiab] or multimedia[tiab]
#10	#8 or #9
#11	COVID-19[mh] or SARS-CoV-2[mh]
#12	covid[tiab] or covid19[tiab] or covid2019[tiab] or "sars-cov-2*"[tiab] or "sarscov-2*"[tiab] or "sars-cov2*"[tiab] or sarscov2*[tiab] or "sars-cov-19"[tiab] or "sarscov-19"[tiab] or "sars-cov19"[tiab] or sarscov19[tiab] or sars2*[tiab] or "2019-novel-cov"[tiab] or "2019-ncov"[tiab] or 2019ncov[tiab] or "ncov-2019"[tiab] or ncov2019[tiab] or coronavirus[tiab] or "corona virus"[tiab]
#13	#11 or #12
#14	#7 and #10 and #13
#15	#14 and ((english[Language]) or (german[Language]))

Table S3. Search strategy for Web of Science Core Collection.

Search	Query
#1	TS=("self-help*" or selfhelp* or "mutual aid" or "mutual help*" or "support group$" or "peer support" or "mutual support" or "group support" or "patient advoca*" or "patient represent*" or "patient organi?ation$" or "patient support organi?ation$" or "patient interest group$" or "patient association$" or "patient support association$" or "health advoca*" or "health interest group$" or "rare disease organi?ation$")
#2	TS=("non-profit organi?ation$" or "nonprofit organi?ation$" or npo$ or "not-for-profit organi?ation$" or nfp$ or "non-government* organi?ation$" or "nongovernment* organi?ation$" or ngo$)
#3	#2 and TS=(health or disease or patient* or advoca* or "self-help*" or selfhelp* or support*)
#4	#1 or #3
#5	TS=(digital* or digitiz* or digitis* or online or "on-line" or webbased or "web-based" or virtual* or cyber or computeri?ed or technological* or electronic* or "e-health" or ehealth or "mobile health" or "m-health" or mhealth or telemedicine or "tele-medicine" or telehealth or "tele-health" or "wearable device$" or internet or web or software or "cell phone$" or "cellular phone$" or "mobile phone$" or smartphone$ or "smart phone$" or "smart device$" or tablet$ or app$ or application$ or "e-mail*" or email* or videoconferenc* or "video-conferenc*" or chat* or "discussion forum$" or "discussion fora" or "social media" or facebook or youtube or whatsapp or instagram or tiktok or twitter or blog* or multimedia or webinar$ or podcast* or webcast*)
#6	TS=(covid or covid19 or covid2019 or "sars-cov-2*" or "sarscov-2*" or "sars-cov2*" or sarscov2* or "sars-cov-19" or "sarscov-19" or "sars-cov19" or sarscov19 or sars2* or "2019-novel-cov" or "2019-ncov" or 2019ncov or "ncov-2019" or ncov2019 or coronavirus or "corona virus")
#7	#4 and #5 and #6
#8	#7 and LA=(English or German)

Table S4. Search strategy for WHO COVID-19 Research Database.

Search	Query
#1	(("self-help" OR "self-helping" OR selfhelp* OR "mutual aid" OR "mutual help" OR "mutual helping" OR "support group" OR "support groups" OR "peer support" OR "mutual support" OR "group support" OR "patient advocacy" OR "patient advocate" OR "patient advocates" OR "patient representative" OR "patient representatives" OR "patient representation" OR "patient organization" OR "patient organizations" OR "patient organisation" OR "patient organisations" OR "patient support organization" OR "patient support

Search	Query
	organizations" OR "patient support organisation" OR "patient support organisations" OR "patient interest group" OR "patient interest groups" OR "patient association" OR "patient associations" OR "patient support association" OR "patient support associations" OR "health advocacy" OR "health advocate" OR "health advocates" OR "health interest group" OR "health interest groups" OR "rare disease organization" OR "rare disease organizations" OR "rare disease organisation" OR "rare disease organisations") OR (("non-profit organization" OR "non-profit organizations" OR "non-profit organisation" OR "non-profit organisations" OR "nonprofit organization" OR "nonprofit organizations" OR "nonprofit organisation" OR "nonprofit organisations" OR npo* OR "not-for-profit organization" OR "not-for-profit organizations" OR "not-for-profit organisation" OR "not-for-profit organisations" OR nfp* OR "non-government organization" OR "non-government organizations" OR "non-government organisation" OR "non-government organisations" OR "nongovernment organization" OR "nongovernment organizations" OR "nongovernment organisation" OR "nongovernment organisations" OR "non-governmental organization" OR "non-governmental organizations" OR "non-governmental organisation" OR "non-governmental organisations" OR "nongovernmental organization" OR "nongovernmental organizations" OR "nongovernmental organisation" OR "nongovernmental organisations" OR ngo*) AND (health OR disease OR patient* OR advoca* OR "self-help" OR "self-helping" OR selfhelp* OR support*)))
#2	(digital* OR digitiz* OR digitis* OR online OR "on-line" OR webbased OR "web-based" OR virtual* OR cyber OR computerized OR computerised OR technological* OR electronic* OR "e-health" OR ehealth OR "mobile health" OR "m-health" OR mhealth OR telemedicine OR "tele-medicine" OR telehealth OR "tele-health" OR "wearable device" OR "wearable devices" OR internet OR web OR software OR "cell phone" OR "cell phones" OR "cellular phone" OR "cellular phones" OR "mobile phone" OR "mobile phones" OR smartphone* OR "smart phone" OR "smart phones" OR "smart device" OR "smart devices" OR tablet* OR app OR apps OR application* OR "e-mail" OR email* OR videoconferenc* OR "video conference" OR "video conferences" OR "video conferencing" OR chat* OR "discussion forum" OR "discussion forums" OR "discussion fora" OR "social media" OR facebook OR youtube OR whatsapp OR instagram OR tiktok OR twitter OR blog* OR multimedia OR webinar* OR podcast* OR webcast*)
#3	(covid OR covid19 OR covid2019 OR "sars-cov-2" OR "sars-cov-2019" OR "sarscov-2" OR "sarscov-2019" OR "sars-cov2" OR "sars-cov2019" OR sarscov2* OR "sars-cov-19" OR "sarscov-19" OR "sars-cov19" OR sarscov19 OR sars2* OR "2019-novel-cov" OR "2019-ncov" OR 2019ncov OR "ncov-2019" OR ncov2019 OR coronavirus OR "corona virus")
#4	#1 AND #2 AND #3
