# Supplementary material for: Patient Organizations’ Digital Responses to the COVID-19 Pandemic: Scoping Review
Source: J Med Internet Res. 2024 Dec 20;26:e58566. doi: 10.2196/58566 (PMC11699494; doi:10.2196/58566)
Supplement: Multimedia Appendix 4 [file jmir_v26i1e58566_app4.pdf]

Multimedia Appendix 4: Characteristics of Included Articles and POs

Table S1. Characteristics of included articles and POs.

Reference	Article Characteristics			PO Characteristics		
	Objective	Methods	Article Type	Name	Indication	Geographic Location
Beck et al. [38]	Evaluate SMART Recovery Australia's scale-up of online support groups during the COVID-19 pandemic.	Mixed methods (e.g., RE-AIM, survey, Zoom data analytics)	Research article	SMART Recovery Australia	Addictive behaviors	Australia
Bergmans et al. [29]	Present lessons learned from transitioning the Skills for Safer Living (SfSL) intervention to a virtual format.	Report	Voices from practice	Canadian Mental Health Association	Mental health	Canada
Bouey et al. [32]	Explore challenges Chinese POs faced during the first COVID-19 lockdown and how they responded.	Mixed methods (survey & focus group)	Research article	Various POs	HIV	China
Chung et al. [37]	Examine the impact of the pandemic on rare disease POs in the Asia-Pacific region.	Mixed methods (survey & focus groups)	Research article	Various POs	Rare diseases	Asia-Pacific region (Australia, China, Hong Kong, India, Japan, Malaysia, New Zealand,

Reference	Article Characteristics			PO Characteristics		
	**Objective**	**Methods**	**Article Type**	**Name**	**Indication**	**Geographic Location**
						Philippines, Singapore, Taiwan)
Constantini et al. [33]	Compare the experiences of members of support groups that have moved to an online format with general findings of the literature on online groups.	Mixed methods (literature search & focus groups)	Research article	PROMENZ	Dementia	Austria
Kelly et al. [30]	Discuss the global impact of COVID-19 on SMART Recovery with facilitators from different regions.	Online exchange between the representatives of different national PO branches from different countries.	Report	SMART Recovery International	Addictive behaviors	International (Affiliations in Australia, Brazil, Denmark, Hong Kong, Ireland, Malaysia, Spain, UK, USA)
Lamont et al. [36]	Explore stroke survivors' perceptions of social support and shared identity within stroke groups during COVID-19 and its impact on psychosocial health.	Survey	Research article	Stroke Association	Stroke	UK

Reference	Article Characteristics			PO Characteristics		
	Objective	Methods	Article Type	Name	Indication	Geographic Location
Marks et al. [35]	Investigate the effectiveness and experiences of participants in online tinnitus support groups and educational webinars implemented during COVID-19.	Interviews	Research article	British Tinnitus Association (now Tinnitus UK)	Tinnitus	UK
McMullan et al. [28]	Examine the impact of the pandemic on rare disease POs in Ireland and the UK.	Survey	Data note	Various POs	Rare diseases	Ireland and UK
Nemeth Blažić et al. [31]	Describe, among other things, the digitalization of a PO's voluntary HIV, HCV, and STI counseling and testing services during the pandemic.	Report	Report	Croatian Association for HIV and Viral Hepatitis	HIV, HCV & other STIs	Croatia
Penfold and Ogden [39]	Explore Gamblers Anonymous members' experiences with online meetings during the pandemic to understand the effectiveness and support provided compared to in-person sessions.	Interviews	Research article	Gamblers Anonymous	Addictive behaviors	UK
Seckam and Hallingberg [34]	Examine stroke survivors' experiences with the transition from live to virtual choir sessions during COVID-19.	Focus groups & interviews	Research article	Stroke Association	Stroke	UK

Reference	Article Characteristics			PO Characteristics		
	Objective	Methods	Article Type	Name	Indication	Geographic Location
Senreich et al. [40]	Explore 12-Step program attendees' experiences with in-person groups transitioning to online meetings.	Focus groups	Research article	Various POs	Addictive behaviors	USA

References

28. McMullan J, Crowe AL, Bailie C, McKnight AJ. Evaluating the impact of COVID-19 on rare disease support groups. BMC Res Notes 2021;14(1):168. PMID:33957984
29. Bergmans Y, Kellington K, Smith T, Pond A, Goving M, Shelton E, Sayegh C, Syms D, Perivolaris A. Providing virtual suicide prevention groups for people experiencing suicidality: Pivoting service delivery during the COVID-19 pandemic. INTERNATIONAL SOCIAL WORK 2021;64(5):801-805. doi:10.1177/0020872821996781
30. Kelly PJ, McCreanor K, Beck AK, Ingram I, O'Brien D, King A, McGlaughlin R, Argent A, Ruth M, Hansen BS, Andersen D, Manning V, Shakeshaft A, Hides L, Larance B. SMART Recovery International and COVID-19: Expanding the reach of mutual support through online groups. J Subst Abuse Treat 2021;131:108568. PMID:34446323
31. Nemeth Blažić T, Bogdanić N, Nola IA, Kosanović Ličina ML, Delaš Aždajić M. Digital technology and HIV, HCV and STI voluntary counselling and testing: good practice example from Croatia. Cent Eur J Public Health 2022;30(2):107-110. PMID:35876599
32. Bouey JZH, Han J, Liu Y, Vuckovic M, Zhu K, Zhou K, Su Y. A case study of HIV/AIDS services from community-based organizations during COVID-19 lockdown in China. BMC Health Serv Res 2023;23(1):288. PMID:36973805
33. Constantini J, Bliem HR, Crepaz M, Marksteiner J. A Qualitative Literature Search and Pilot Study of Online Support Groups for Patients With Dementia and Their Carers. Gerontol Geriatr Med 2023;9:23337214231205689. PMID:37881348
34. Seckam A, Hallingberg B. The experiences and perceptions of stroke survivors engaging in a virtual choir during COVID-19: a thematic analysis. British Journal of Neuroscience Nursing 2021;17(Sup5):S18-S25. doi:10.12968/bjnn.2021.17.Sup5.S18
35. Marks E, Handscomb L, Remskar M. "I can see a path forward now": a qualitative investigation of online groups for tinnitus in the time of Covid-19. Int J Audiol 2022:1-8. PMID:35499467
36. Lamont RA, Calitri R, Mounce LTA, Hollands L, Dean SG, Code C, Sanders A, Tarrant M. Shared social identity and perceived social support among stroke groups during the COVID-19 pandemic: Relationship with psychosocial health. Appl Psychol Health Well Being 2022. PMID:35139581
37. Chung CCY, Ng YNC, Jain R, Chung BHY. A thematic study: impact of COVID-19 pandemic on rare disease organisations and patients across ten jurisdictions in the Asia Pacific region. Orphanet J Rare Dis 2021;16(1):119. PMID:33673852
38. Beck AK, Larance B, Baker AL, Deane FP, Manning V, Hides L, Kelly PJ. Supporting people affected by problematic alcohol, substance use and other behaviours under pandemic conditions: A pragmatic evaluation of how SMART recovery Australia responded to COVID-19. Addict Behav 2023;139:107577. PMID:36528964
39. Penfold KL, Ogden J. Exploring the experience of Gamblers Anonymous meetings during COVID-19: a qualitative study. Curr Psychol 2022;41(11):8200-8213. PMID:34421284

40. Senreich E, Saint-Louis N, Steen JT, Cooper CE. The Experiences of 12-Step Program Attendees Transitioning to Online Meetings during the COVID-19 Pandemic. Alcoholism Treatment Quarterly 2022;40(4):500-517. doi:10.1080/07347324.2022.2102456
