# Supplementary material for: Patient Organizations’ Digital Responses to the COVID-19 Pandemic: Scoping Review
Source: J Med Internet Res. 2024 Dec 20;26:e58566. doi: 10.2196/58566 (PMC11699494; doi:10.2196/58566)
Supplement: Multimedia Appendix 5 [file jmir_v26i1e58566_app5.pdf]

Multimedia Appendix 5: Consolidated Findings on POs' Digital Adaptations of Group-Based Support Activities

Table S1. Characteristics of digital adaptations of group-based support activities.

Reference	Services/ Activities	Digital Technologies	Description
Beck at al. [38]	Support groups	Zoom	To meet support needs during the COVID-19 pandemic, SMART Recovery Australia expanded its online support groups from 6 to 132 with funding from the Commonwealth Government of Australia. The initiative aimed to establish more than 100 online groups within a year, with 80% managed by third-party providers to increase sustainability. The expansion provided facilitators with online group management tools, including PowerPoint presentations and a Facebook group for networking. Training was provided through a dedicated online platform developed in 2019, which offered video role-playing and skill-building sessions. In addition, SMART Recovery Australia hosted regular Zoom meetings for facilitators to share best practices and foster community.
Bergmans et al. [29]	Psychosocial/ psychoeducational group intervention	Microsoft Teams, PowerPoint, Paint, videos, digital games	One month into the pandemic, the team pivoted to deliver Skills for Safer Living (SfSL) groups virtually. They piloted three 8-week virtual sessions using Microsoft Teams, each with three facilitators for comprehensive support. Preparation included weekly team meetings and facilitators familiarizing themselves with Microsoft Teams and complementary applications such as PowerPoint, digital games, and screen sharing to enhance group interaction and facilitate dynamic sessions. Online delivery involved three facilitators per group to ensure comprehensive support and to meet both the technological and therapeutic needs of the participants.

Reference	Services/ Activities	Digital Technologies	Description
Constantini et al. [33]	Support groups	Zoom	Transition of support groups to online platforms, primarily Zoom.[a]
Kelly et al. [30]	Support groups	Online (not specified)	SMART Recovery International rapidly transitioned to online mutual support groups, maintaining the same format and approach as their in-person meetings. Prior to the pandemic, there were a limited number of online groups in the United States, England, and Australia. During the pandemic, these numbers increased, with SMART Recovery USA expanding from 40 to over 1,200 groups. In regions without existing online groups, such as Denmark, Ireland, Hong Kong, Spain, Malaysia, and Brazil, international collaboration was critical to establishing these groups for the first time.
Lamont et al. [36]	Various support groups	Email, text messaging, video calls, social media	Several Stroke Association peer support groups with activities such as 'support', 'speakability/aphasia', or 'creative arts', stayed in touch through digital methods like video calls, email, and text messaging. Although they maintained contact, the original core activities were not necessarily directly adapted.[a]
Marks et al. [35]	Tinnitus support groups	Zoom	The British Tinnitus Association (BTA) developed online support groups that were conducted via Zoom. These groups were hosted monthly for 1.5 hours by various experienced BTA volunteers. These groups were open to an unlimited number of participants and typically attracted around 15 participants per session. The content varied, with either an invited speaker or a specific topic for discussion. These free groups were advertised and could be booked through the BTA website and were managed by BTA staff for technical support.[a]
Penfold and Ogden [39]	Support groups	Zoom	Gamblers Anonymous established online meetings within a few weeks, accessible to members and conducted via Zoom.[a]

Reference	Services/ Activities	Digital Technologies	Description
Seckam and Hallingberg [34]	Choir	Zoom	The live Strike a Chord choir of the Stroke Association Wales transitioned to a virtual environment using Zoom. Each session incorporated a 10-minute socialization period before singing, allowing participants to interact and connect.[a]
Senreich et al. [40]	Support groups	Video-conferencing (primarily via Zoom)	Various 12-step programs rapidly transitioned their meetings to an online format at the onset of the COVID-19 pandemic.[a]

[a] Note that this study focused primarily on participants' experiences rather than on the details of the digital adaptations.

Table S2. Positive outcomes of digital adaptations of group-based support activities.

Reference	Thematic Code	Description
**Adaptation & Execution**		
Bergmans et al. [29]	Continuity of Support	The digital adaptation of the SfSL groups enabled the ongoing provision of vital support services for high-risk individuals.
Constantini et al. [33]	Continuity of Group Support	Digital transformation enabled support groups to continue during the pandemic.
Kelly et al. [30]	Continuity of Group Support	Rapid digital expansion maintained the continuity of mutual support during the pandemic.
Senreich et al. [40]	Easier Online Service Execution	Online meetings simplified the process of conducting meetings, eliminating physical tasks like setting up the room or making coffee.
	Positive Response to Transition	Participants appreciated the quick and effective transition to online meetings at the onset of the pandemic.

Reference	Thematic Code	Description
**Accessibility**		
Bergmans et al. [29]	Improving Accessibility	The online format removed previous access barriers (e.g., transportation and health issues).
Kelly et al. [30]	Improving Accessibility	The expansion of online groups provided broader access (e.g., in areas where in-person meetings were not feasible).
Marks et al. [35]	Improving Accessibility	Participants valued the nationwide accessibility of online formats, especially beneficial for those with travel restrictions due to work, health, or other reasons, and wished for these formats to continue after the pandemic.[a]
Penfold and Ogden [39]	Removal of Geographical Barriers	Online meetings expanded accessibility by removing geographical boundaries, allowing participation from any location.
	Broadened Meeting Selection	The shift to online enabled participation in a wider selection of meetings, including various types, both locally and internationally.
Senreich et al. [40]	Improving Accessibility	Online meetings improved access in areas where in-person options are generally limited.
	Global Access & Diversity	By moving online, 12-step meetings became accessible worldwide at any time of the day, resulting in a greater variety of meetings.
**Participant Engagement & Perception**		
Beck at al. [38]	Positive Perception of Online Group Delivery	The majority found online group meetings well facilitated and helpful.
	High Preference for Online Meetings	Among participants who experienced both formats, half preferred online meetings to face-to-face meetings, while more than one-third had no preference.

Reference	Thematic Code	Description
	Helpful Recovery Contribution	The majority left online meetings with practical recovery information and resources.
Bergmans et al. [29]	Feeling of Emotional Safety	Providing virtual groups created a sense of emotional safety for some participants.
Lamont et al. [36]	Maintaining Group Connections	Among the respondents to the survey, the majority maintained contact with their group throughout the pandemic, predominantly via online tools and phone.
Marks et al. [35]	Preference for Online Groups	Some participants preferred online support groups, finding them to be an improvement over in-person sessions.
	Ease of Information Sharing	The digital format made it easy to share tips, increasing the usefulness of post-session strategies.[a]
	High Value of Online Groups	Participants seeking support during the pandemic valued the availability of online groups.[a]
Penfold and Ogden [39]	Novelty & Variety	The shift to online meetings brought a sense of novelty and variety that made the experience enjoyable and uplifting.
	Impact of Positive Self-Concept on Attendance	The meetings helped participants develop a more positive perception of themselves, which was an important factor in motivating them to continue attending.
**Interpersonal Dynamics**		
Beck at al. [38]	Feeling of Inclusion	The majority of participants felt welcome in online group meetings.
	Feeling Supported	The majority of participants felt supported in the online group meetings.
Bergmans et al. [29]	Presence of Therapeutic Interpersonal Elements	Therapeutic elements of group intervention, including universality, information sharing, altruism, belonging, and catharsis, were experienced in online formats.

Reference	Thematic Code	Description
Constantini et al. [33]	Emotional Capacity & Connectivity via Videoconference	Videoconferencing enabled intimate group meetings in which participants engaged emotionally and felt connected with each other, despite being diagnosed with mild to moderate dementia.
Lamont et al. [36]	High Social identification	During the pandemic, members' strong identification with their group, maintained through engagement via online tools and phone, was associated with positive psychosocial outcomes.
Marks et al. [35]	Presence of Therapeutic Interpersonal Elements	Key therapeutic elements such as social connectedness, reduced isolation, and shared learning were experienced through online formats.[a]
Penfold and Ogden [39]	Presence of Interpersonal Aspects	Online meetings preserved key aspects of the group experience, such as social comparison, affirmation, support networks, and feelings of solidarity and togetherness.
	Nurturing Togetherness and Collectivity	Online meetings were perceived as nurturing spaces that fostered togetherness and collective identity.
Seckam and Hallingberg [34]	Social Connectivity	Virtual choir sessions provided participants with a sense of belonging, maintained vital social connections, and helped combat feelings of social isolation.
	Emotional Uplift from Virtual Meetings	Participants reported feeling uplifted, empowered and inspired by virtual choir sessions.
Senreich et al. [40]	Enhanced Social Interaction in Online Environments	The online format and features such as chat boxes made meeting people easier and less intimidating for some participants.
**Privacy & Security**		
Kelly et al. [30]	Increased Anonymity	Online meetings have created new opportunities for anonymity, such as turning off the camera, which can reduce the stigma associated with participation for some people.

Reference	Thematic Code	Description
**Future & Continuation**		
Beck at al. [38]	Continuation of Online Groups	The majority of participants intended to continue with the online groups.
Constantini et al. [33]	Continuation of Online Groups	Many participants wanted to continue with online groups after the pandemic-related restrictions were lifted.
Kelly et al. [30]	Validation of Online Support	The digital transformation during COVID-19 confirmed to the SMART Recovery community (members, facilitators, and administrators) that online groups are a suitable alternative to in-person meetings.
	Lasting Online Infrastructure Development	As a positive outcome of the pandemic, the infrastructure and expertise necessary to conduct online SMART Recovery groups has been established, making ongoing online groups possible.
Marks et al. [35]	Continuation of Online Groups	Many participants expressed a desire for the online groups to continue after the pandemic, recognizing the relevance of digital approaches in the modern world.[a]
Seckam and Hallingberg [34]	Yearning for Face-to-Face Interaction	While embracing the virtual sessions, participants expressed a longing for face-to-face choir meetings.
Senreich et al. [40]	Continuation of Online Groups & Dismantling of Stigmas	During the pandemic, the stigma associated with online meetings was reduced as participants now expect them to be a routine option after the pandemic.

[a] Note that in presenting their findings, Marks et al. [35] did not consistently differentiate between the experiences of workshop participants and those of support group participants.

Table S3. Challenges and barriers of digital adaptations of group-based support activities.

Reference	Thematic Code	Description
**Adaptation & Execution**		
Bergmans et al. [29]	Adaptation Challenges due to Lack of Established Guidance	Adapting suicide prevention groups and their therapeutic aspects to a virtual environment presented challenges, as the delivery needed to be reimagined, particularly due to the lack of specific guidance on conducting online sessions for individuals with experiences of suicidality.
Kelly et al. [30]	Lack of Pre-existing Online Groups	The transition was challenging in countries without pre-existing online groups, as digital infrastructure had to be newly established and facilitators trained.
	High Costs of Online Groups	Facilitating online mutual support is costly (e.g., due to licenses and digital equipment).
Senreich et al. [40]	Difficulty Replicating In-Person Formats	Converting well-established face-to-face practices such as "round robin" meetings or abstinence anniversary celebrations to Zoom was a challenge.
	Reduced Willingness to Volunteer Online	Decreased willingness to volunteer for service roles in online meetings.
**Accessibility**		
Beck at al. [38]	Technical Difficulties during Meetings	One in five participants reported technical difficulties during online meetings.
Constantini et al. [33]	Technical Difficulties	Participants faced challenges with inconsistent Internet connections during online meetings.
Lamont et al. [36]	Technology Barriers (Availability & Skills)	For some members, participation was challenging due to a lack of access to technology or difficulties using it.

Reference	Thematic Code	Description
	Health-Related Barriers	Stroke-related disabilities posed barriers to maintaining contact with the group (via online tools and telephone), often necessitating reliance on family or caregiver support.
Marks et al. [35]	Initial Technological Barriers	For some participants, the use of technology was initially challenging (participants overcame this with facilitator support).[a]
Seckam and Hallingberg [34]	Technology Barriers (Availability & Skills)	Lack of access to essential technology and/or limited ability to use it led to the exclusion of certain members.
Senreich et al. [40]	Technology Barriers (Availability & Skills)	Participants noted that those less familiar with digital technology had difficulty participating in or were excluded from online meetings, as were homeless individuals without access to technology.
	Meeting Information Accuracy	Many participants expressed frustration with difficulties accessing Zoom meetings due to missing or incorrect information (e.g., passcodes).
**Participant Engagement & Perception**		
Constantini et al. [33]	Reduced Physical Activity	The shift to online groups raised concerns about reduced physical activity among participants.
Penfold and Ogden [39]	Dropouts	The transition to online meetings resulted in dropouts due to members' reluctance to adapt to the new format.
	Zoom Fatigue	The increased use of online tools for daily activities during the pandemic led to a decrease in motivation to attend online group meetings.
	Dropouts	Some group members chose not to continue attending meetings after the shift to an online format.
Seckam and Hallingberg [34]	Limitations of Virtual Experience	For some, the virtual choir did not replicate the in-person experience.
	Reduced Physical Activity	Reduced physical activity was seen by some as a disadvantage of the virtual choir, as participants remained seated during sessions.

Reference	Thematic Code	Description
Senreich et al. [40]	Negative Impact on Recovery	Several participants felt that this format was not always beneficial to their recovery, citing feelings of laziness and isolation.
**Interpersonal Dynamics**		
Lamont et al. [36]	Lack of Close Pre-Pandemic Relationships	Several members reported that not knowing the group well or not having a close relationship with other members before the pandemic was a barrier to staying in touch during the pandemic.
Marks et al. [35]	Social Comparisons' Negative Impact	Observing others' progress led to feelings of jealousy, and seeing struggles provoked fear in some participants.[a]
	Lack of Informal Communication	The absence of informal chat in online sessions affected relationship building.[a]
	Lack of Physical Presence	A minority of participants experienced a reduced sense of connection online compared to in-person interactions.[a]
Penfold and Ogden [39]	Lack of Physical Presence	Online meetings could not fully capture the emotional connection of being physically together.
	Lack of Solidarity	The shift to online meetings has affected group solidarity, as some members have been rejected by others for not wanting to participate in an online format.
Senreich et al. [40]	Challenges in Online Fellowship	The virtual format presented challenges in offering support to distressed individuals or newcomers, as well as in establishing sponsor-sponsee relationships.
	Lack of Human Connection in Online Meetings	Participants found that online meetings could not fully capture the human connection and energy of in-person meetings, missing elements such as physical contact and social interaction before and after meetings.

Reference	Thematic Code	Description
**Privacy & Security**		
Bergmans et al. [29]	Challenges of Managing Privacy	Managing privacy in virtual settings was experienced as a challenge, as facilitators had less control over it.
Penfold and Ogden [39]	Jeopardy of Anonymity	Participants expressed concern that online meetings would compromise the essential principle of anonymity that is critical to Gamblers Anonymous meetings. Turning off cameras was one way to regain anonymity, while others preferred that all participants leave their cameras on.
Senreich et al. [40]	Incidents of "Zoom Bombing"	Some participants reported incidents of "Zoom bombing," where unauthorized individuals disrupted meetings with hateful comments.

[a] Note that in presenting their findings, Marks et al. [35] did not consistently differentiate between the experiences of workshop participants and those of support group participants.

Table S4. Facilitating factors of digital adaptations of group-based support activities.

Reference	Thematic Code	Description
**Adaptation & Execution**		
Beck at al. [38]	Government Funding	Upscaling of online mutual support has been supported by government funding.
Bergmans et al. [29]	Utilization of Webcams and Non-Verbal Cues	Webcams and gestures improved communication and helped in reading body language, increasing group cohesion in virtual meetings.
	Additional Facilitator as Supportive Structure	An additional facilitator monitored non-verbal cues, offered tech support, and provided additional support (e.g., in crisis situations).
	Addressing Technological Concerns in Pre-Session	Pre-group 'coaching' sessions effectively helped clients understand and navigate the virtual format, mitigating technical concerns and thereby

Reference	Thematic Code	Description
		improving the overall quality of online meetings (e.g., regarding audio and visual quality).
	Utilization of Various Forms of Content Delivery	Using various digital tools to share content kept participants engaged.
	Flexibility of Facilitators	The facilitators' flexibility in scheduling was crucial, as demonstrated when some seamlessly filled in for others on personal leave, ensuring the smooth continuation of group facilitation and support.
Constantini et al. [33]	Various Principles Relevant for Successful Transformation	Creativity, technical savvy, and motivation were highlighted as principles for transitioning self-help groups into virtual spaces.
	Skilled Facilitator	An experienced facilitator was crucial in guiding the group members, especially at the beginning of the digital adaptation.
Kelly et al. [30]	Government Funding	The transition of mutual support groups to an online format was supported by government funding.
	Pre-Existing Online Experience & International Collaboration	International collaboration to share experiences and resources facilitated the establishment of online groups in countries where they did not exist before the pandemic.
Marks et al. [35]	Use of Breakout Rooms	Breakout rooms enabled more effective interaction within smaller groups.[a]
	Skilled Facilitators	Professional facilitators contributed to the sessions being perceived as reliable, diverse, efficient, supportive, and dynamic. This professional involvement fostered trust, hope, motivation, and engagement among theparticipants.[a]
Seckam and Hallingberg [34]	Pre-Experiences with Digital Platforms	The participants' previous experience with digital communication made the transition to using Zoom for virtual choir sessions easy for most.

Reference	Thematic Code	Description
**Interpersonal Dynamics**		
Bergmans et al. [29]	Making Room for Social Interactions	Setting aside time before and after online sessions encouraged social engagement and mimicked the dynamics of in-person meetings.
**Privacy & Security**		
Bergmans et al. [29]	Addressing Privacy Concerns in Pre-Session	Facilitators conducted pre-group 'coaching' sessions, which helped participants understand and navigate the privacy settings of virtual formats, thereby helping to mitigate privacy concerns.

[a] Note that in presenting their findings, Marks et al. [35] did not consistently differentiate between the experiences of workshop participants and those of support group participants.

References

29. Bergmans Y, Kellington K, Smith T, Pond A, Goving M, Shelton E, Sayegh C, Syms D, Perivolaris A. Providing virtual suicide prevention groups for people experiencing suicidality: Pivoting service delivery during the COVID-19 pandemic. INTERNATIONAL SOCIAL WORK 2021;64(5):801-805. doi:10.1177/0020872821996781
30. Kelly PJ, McCreanor K, Beck AK, Ingram I, O'Brien D, King A, McGlaughlin R, Argent A, Ruth M, Hansen BS, Andersen D, Manning V, Shakeshaft A, Hides L, Larance B. SMART Recovery International and COVID-19: Expanding the reach of mutual support through online groups. J Subst Abuse Treat 2021;131:108568. PMID:34446323
33. Constantini J, Bliem HR, Crepaz M, Marksteiner J. A Qualitative Literature Search and Pilot Study of Online Support Groups for Patients With Dementia and Their Carers. Gerontol Geriatr Med 2023;9:23337214231205689. PMID:37881348
34. Seckam A, Hallingberg B. The experiences and perceptions of stroke survivors engaging in a virtual choir during COVID-19: a thematic analysis. British Journal of Neuroscience Nursing 2021;17(Sup5):S18-S25. doi:10.12968/bjnn.2021.17.Sup5.S18
35. Marks E, Handscomb L, Remskar M. "I can see a path forward now": a qualitative investigation of online groups for tinnitus in the time of Covid-19. Int J Audiol 2022:1-8. PMID:35499467
36. Lamont RA, Calitri R, Mounce LTA, Hollands L, Dean SG, Code C, Sanders A, Tarrant M. Shared social identity and perceived social support among stroke groups during the COVID-19 pandemic: Relationship with psychosocial health. Appl Psychol Health Well Being 2022. PMID:35139581
38. Beck AK, Larance B, Baker AL, Deane FP, Manning V, Hides L, Kelly PJ. Supporting people affected by problematic alcohol, substance use and other behaviours under pandemic conditions: A pragmatic evaluation of how SMART recovery Australia responded to COVID-19. Addict Behav 2023;139:107577. PMID:36528964
39. Penfold KL, Ogden J. Exploring the experience of Gamblers Anonymous meetings during COVID-19: a qualitative study. Curr Psychol 2022;41(11):8200-8213. PMID:34421284
40. Senreich E, Saint-Louis N, Steen JT, Cooper CE. The Experiences of 12-Step Program Attendees Transitioning to Online Meetings during the COVID-19 Pandemic. Alcoholism Treatment Quarterly 2022;40(4):500-517. doi:10.1080/07347324.2022.2102456
