# Supplementary material for: Patient Organizations’ Digital Responses to the COVID-19 Pandemic: Scoping Review
Source: J Med Internet Res. 2024 Dec 20;26:e58566. doi: 10.2196/58566 (PMC11699494; doi:10.2196/58566)
Supplement: Multimedia Appendix 6 [file jmir_v26i1e58566_app6.pdf]

Multimedia Appendix 6: Consolidated Findings on POs' Digital Adaptations of Communication, Counseling, Information Provision, Educational Activities, and Other Operational Aspects

Table S1. Characteristics of digital adaptations of communication, counseling, information provision, educational activities, and other operational aspects.

Reference	Services/ Activities	Digital Technologies	Description
Bouey et al. [32]	HIV counseling & testing	WeChat, TikTok, other platforms (not further specified)	During the pandemic, many Chinese POs supporting people living with HIV shifted aspects of their face-to-face services, such as HIV counseling and testing, to online (and telephone-based) platforms. POs used various digital platforms such as WeChat, TikTok, their website, and other online tools to manage service requests. These platforms not only facilitated communication, but also the organization of services such as testing appointments.[a]
Chung et al. [37]	Communication, counseling, information provision	Social media, discussion forums, messenger (e.g., WhatsApp), video calls (e.g. Zoom, Skype), email, newsletter, website	POs used digital platforms and tools, such as social media and video calls, to stay in touch, provide support (e.g., through group chat, Facebook groups, discussion forums, or online counseling), and share information with patients via email updates/newsletters or website.[a]

Reference	Services/ Activities	Digital Technologies	Description
Marks et al. [35]	Tinnitus educational workshops	Zoom	In response to the pandemic, the British Tinnitus Association (BTA) developed online educational workshops. These workshops, facilitated by an experienced therapist, consisted of two 2-hour sessions one to two weeks apart. Each workshop was designed to accommodate up to 12 participants, with typically 3-5 participants attending the first session alone. They were conducted using Zoom, had a fee of £5, and were advertised and booked through the BTA website.[b]
McMullan et al. [28]	Communication, information provision, committee meetings	Zoom, newsletters, webinars	In response to the COVID-19 pandemic, rare disease POs adapted their communication methods using digital tools. This shift included holding virtual meetings, facilitating chats, publishing e-newsletters, and hosting webinars.[a]
Nemeth Blažić et al. [31]	HIV/STIs counseling & testing	Data management software, email	The CheckPoint Centre Zagreb of the Croatian Association for HIV and Viral Hepatitis (CAHIV) digitalized various aspects of its voluntary counseling and testing (VCT) services for HIV, hepatitis C, and other STIs during the pandemic to minimize face-to-face contact. They developed software to manage client data and test results and upgraded an existing application. The counseling process was moved to email or telephone, while maintaining on-site sample collection. Test results, along with additional post-counseling, were provided via email or phone.[c]

[a] Note that this study explored the overall impact of the COVID-19 pandemic on POs, without focusing exclusively on their digital adaptations.

[b] Note that this study focused primarily on participants' experiences rather than on the details of the digital adaptations.

[c] Note that this article highlights examples of good practice in the use of digital technologies for VCT for HIV and STIs. The response to the COVID-19 pandemic is only one of the aspects reported.

Table S2. Positive outcomes of digital adaptations of communication, counseling, information provision, educational activities, and other operational aspects.

Reference[a]	Thematic Code	Description
**Adaptation & Execution**		
Bouey et al. [32]	Successful Adaptation	Some POs described the ability to provide online services (e.g., online counseling) as a key capacity needed during the pandemic.
Chung et al. [37]	Successful Adaptation	Half of the organizations that reported successes during the pandemic attributed their successes to efficiently conducting meetings, consultations, and sharing information with members through online platforms. This shift to digital was a key factor in the perceived success of many organizations during the pandemic.
	Geographic Disparities in Success in Digitalizing Operations	The study revealed significant differences in the response of rare disease POs across various countries to the pandemic. While all organizations in Australia and New Zealand successfully adapted and digitized their operations, others, such as those in Hong Kong, struggled, possibly due to a lack of technological infrastructure or prior experience with online operations.
McMullan et al. [28]	Building an Online Community	The transition to online platforms resulted in the creation of an online community.
	Remote Working	Remote working was reported as a positive impact of the pandemic on operational aspects.
**Accessibility**		
Marks et al. [35]	Improving Accessibility	Participants valued the nationwide accessibility of online formats, especially beneficial for those with travel restrictions due to work, health, or other reasons, and wished for these formats to continue after the pandemic.[b]

Reference[a]	Thematic Code	Description
McMullan et al. [28]	Improving Accessibility	The shift to online communication eliminated the need for physical presence, making POs more accessible to people with mobility or financial limitations.
**Participant Engagement & Perception**		
Marks et al. [35]	Ease of Information Sharing	Participants appreciated the digital format, which facilitated the sharing of tips and increased the usefulness of post-session strategies.[b]
	High Value of Online Formats	Participants seeking support during the pandemic valued the availability of online formats.[b]
**Interpersonal Dynamics**		
Marks et al. [35]	Presence of Therapeutic Interpersonal Elements	Key therapeutic elements such as social connectedness, reduced isolation, and shared learning were experienced in online formats.[b]
**Future & Continuation**		
Marks et al. [35]	High Continuation Interest	Many participants expressed a strong interest in extending the workshop beyond the initial two sessions, recognizing the relevance of digital approaches in the modern world.

[a] Nemeth Blažić et al. [31] did not provide information on 'positive outcomes'.
[b] Note that in presenting their findings, Marks et al. [35] did not consistently differentiate between the experiences of workshop participants and those of support group participants.

Table S3. Challenges and barriers of digital adaptations of communication, counseling, information provision, educational activities, and other operational aspects.

Reference[a]	Thematic Code	Description
**Adaptation & Execution**		
Bouey et al. [32]	Unreliable Digital Infrastructure	POs reported challenges with unreliable Wi-Fi connectivity.

Reference[a]	Thematic Code	Description
Chung et al. [37]	Lack of Digital Infrastructure	Difficulty to digitally adapt operations due to technological limitations in some regional areas.
	Varied Digital Comfort & Skills Among Members	Supporting patients became a challenge with the reliance on online communication, as members had varying levels of comfort and competence with technology.
**Accessibility**		
Marks et al. [35]	Initial Technological Barriers	For some participants, the use of technology was initially challenging (participants overcame this with facilitator support).[b]
**Interpersonal Dynamics**		
Marks et al. [35]	Social Comparisons' Negative Impact	Observing others' progress led to feelings of jealousy, and seeing struggles provoked fear in some participants.[b]
	Lack of Informal Communication	The absence of informal chat in online sessions affected relationship building.[b]

[a] McMullan et al. [28] and Nemeth Blažić [31] did not provide information on 'challenges and drawbacks'.
[b] Note that in presenting their findings, Marks et al. [35] did not consistently differentiate between the experiences of workshop participants and those of support group participants.

Table S4. Facilitating factors of digital adaptations of communication, counseling, information provision, educational activities, and other operational aspects.

Reference[a]	Thematic Code	Description
**Adaptation & Execution**		
Chung et al. [37]	Pre-existing Digitalization	Organizations with pre-existing digital operations, especially in regions such as Australia and New Zealand, adapted more easily.
Marks et al. [35]	Use of Breakout Rooms	Breakout rooms enabled more effective interaction within smaller groups.[b]

Reference[a]	Thematic Code	Description
	Skilled Facilitators	Professional facilitators contributed to the sessions being perceived as reliable, diverse, efficient, supportive, and dynamic. This professional involvement fostered trust, hope, motivation, and engagement among the participants.[b]

[a] Bouey et al. [32], McMullan et al. [28] and Nemeth Blažić et al. [31] did not provide information on 'facilitating factors'.

[b] Note that in presenting their findings, Marks et al. [35] did not consistently differentiate between the experiences of workshop participants and those of support group participants.

References

28. McMullan J, Crowe AL, Bailie C, McKnight AJ. Evaluating the impact of COVID-19 on rare disease support groups. BMC Res Notes 2021;14(1):168. PMID:33957984
31. Nemeth Blažić T, Bogdanić N, Nola IA, Kosanović Ličina ML, Delaš Aždajić M. Digital technology and HIV, HCV and STI voluntary counselling and testing: good practice example from Croatia. Cent Eur J Public Health 2022;30(2):107-110. PMID:35876599
32. Bouey JZH, Han J, Liu Y, Vuckovic M, Zhu K, Zhou K, Su Y. A case study of HIV/AIDS services from community-based organizations during COVID-19 lockdown in China. BMC Health Serv Res 2023;23(1):288. PMID:36973805
35. Marks E, Handscomb L, Remskar M. "I can see a path forward now": a qualitative investigation of online groups for tinnitus in the time of Covid-19. Int J Audiol 2022:1-8. PMID:35499467
37. Chung CCY, Ng YNC, Jain R, Chung BHY. A thematic study: impact of COVID-19 pandemic on rare disease organisations and patients across ten jurisdictions in the Asia Pacific region. Orphanet J Rare Dis 2021;16(1):119. PMID:33673852
